# Long‐Term Outcome After Pulsed Field Ablation for Atrial Fibrillation Across an Atrial Septal Defect Closure Device: A 20‐Month Follow‐Up Case Report

**DOI:** 10.1002/ccr3.73491

**Published:** 2026-09-07

**Authors:** Ahmed Abdelrazik, Ibrahim Antoun, Ivelin Koev, Mahmoud Eldesouky, Marinos Kantzis, G. Andre Ng

**Affiliations:** ^1^ Department of Cardiology University Hospitals of Leicester NHS Trust, Glenfield Hospital Leicester UK; ^2^ Division of Cardiovascular Sciences Clinical Science Wing, University of Leicester, Glenfield Hospital Leicester UK; ^3^ National Institute for Health Research, Leicester Biomedical Research Centre Leicester UK; ^4^ Leicester British Heart Foundation Centre of Research Excellence Leicester UK

**Keywords:** atrial fibrillation, atrial septal defect, case report, pulmonary vein isolation, pulsed‐field ablation

## Abstract

Balloon‐assisted transseptal puncture across a large atrial septal defect closure device can permit pulsed‐field ablation in selected patients with complex septal anatomy, achieving durable pulmonary vein isolation and sinus rhythm. A borderline residual left‐to‐right shunt developed post‐procedure but decreased on serial imaging without hemodynamic consequence.

## 1 Introduction

Atrial fibrillation (AF) is the most common sustained arrhythmia and is frequently encountered in adults with congenital heart disease, including patients with repaired or unrepaired atrial septal defect (ASD) [1, 2, 3]. In ASD, chronic right‐sided volume loading, atrial stretch, and atrial structural and electrical remodeling may create an arrhythmogenic substrate that can persist even after defect closure. Contemporary AF guidelines support rhythm‐control strategies in symptomatic patients, particularly when symptoms remain substantial despite medical therapy or when restoration of sinus rhythm may improve functional capacity and quality of life [4].

Catheter ablation in patients with ASD closure devices can be technically challenging because the occluder may occupy most of the interatrial septum, limiting access to native septal tissue and complicating safe transseptal puncture. Previous reports have described intracardiac echocardiography‐ or transoesophageal echocardiography‐guided puncture through residual native septum or directly through septal occluder devices, with progressive tract dilatation or balloon assistance used when sheath passage is difficult [5, 6, 7, 8, 9, 10]. However, most published experience has involved radiofrequency ablation, and evidence regarding pulsed‐field ablation (PFA) in this setting remains limited.

PFA is a non‐thermal ablation modality based on irreversible electroporation. Its relative myocardial selectivity and reduced collateral thermal injury may be advantageous in anatomically complex patients, including selected adults with congenital heart disease [11]. Nevertheless, PFA systems may require large‐bore delivery sheaths and create a specific access challenge when crossing a large septal occluder device.

We describe a case of persistent AF ablation using PFA in a patient with a large ASD closure device and very limited residual native septal tissue. Balloon dilatation of the device tract enabled left‐atrial access and delivery of PFA, while careful imaging and fluoroscopic guidance were used to avoid device interaction. The case is clinically relevant because it combines a complex transseptal access strategy, use of PFA across an occluder device, and 20‐month longitudinal follow‐up demonstrating durable sinus rhythm and regression of a borderline residual interatrial shunt.

## 2 Case History

We present the case of a 51‐year‐old woman with a history of ASD requiring transcatheter closure in 2018 using a 33‐mm Figulla Flex II device (Occlutech, Switzerland). Her primary symptoms of dizziness and exertional shortness of breath improved after ASD closure. Follow‐up echocardiograms at 2 and 10 months showed the device in situ with no residual shunting. The right ventricle (RV) remained mildly to moderately dilated with preserved function. Her medical history included hypothyroidism (on Levothyroxine), hypertension, treated depression, and prior back surgery for sciatica with ongoing symptoms.

The patient was referred to our arrhythmia clinic in 2023 after struggling with AF for 3 years and failing two direct current cardioversion (DCCV) attempts on Amiodarone. Since developing persistent AF, she reported a significant decline in exercise capacity with fatigue, palpitations, breathlessness, and dizziness. She also recalled paroxysmal palpitations and dyspnoea before ASD closure. After explaining the likely anatomical challenges and the standard success rates for persistent AF ablation, the patient consented to proceed to restore SR.

### 2.1 Investigations and Treatment

**FIGURE 1:**
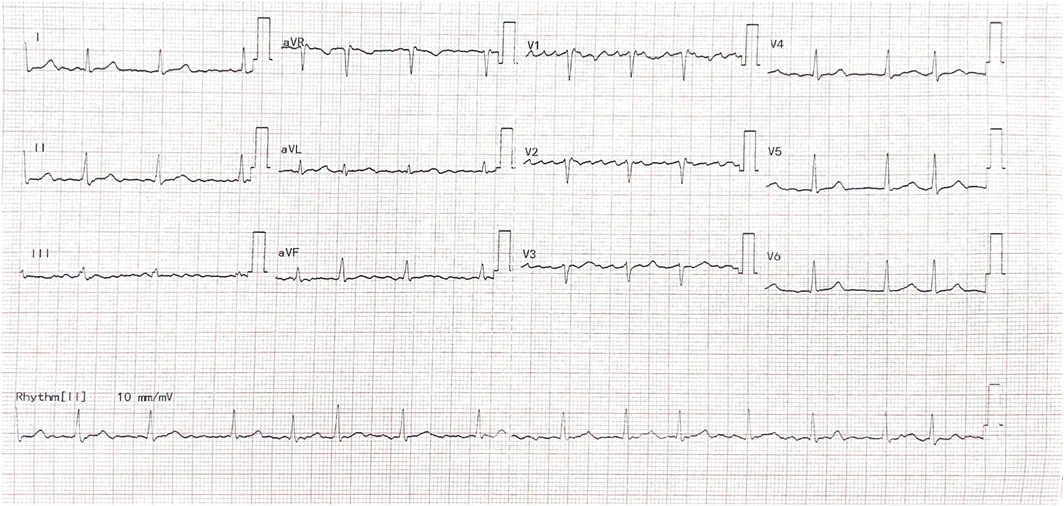
Pre‐assessment ECG showing atrial fibrillation (AF).

Pre‐procedural assessment ECG revealed AF with a controlled ventricular rate of 90 bpm, normal axis, and no signs of ischaemia or pre‐excitation (Figure 1). Echocardiography performed in February 2023 confirmed the ASD device in situ, with good biventricular function. Blood pressure was well controlled, and blood investigations were unremarkable.

The procedure was performed under general anesthesia (GA) and transoesophageal echocardiography (TOE) guidance in February 2024. TOE was used to define the relationship between the ASD closure device and the interatrial septum, to guide transseptal access, and to monitor for acute complications. Right femoral venous access was obtained, and 6F, 7F, and 8F sheaths were secured. A decapolar catheter was positioned in the coronary sinus (CS) and a quadripolar catheter in the right ventricle. TOE demonstrated very limited residual native septal tissue behind the large ASD device. Transseptal puncture was therefore attempted through the device using a Brockenbrough needle. Although left atrial access was obtained, the LAMP‐90 sheath could not be advanced. Sequential dilation using a 16F dilator and Agilis sheath allowed entry into the left atrium; however, the FARADRIVE sheath could not be advanced, despite supportive wire position.

**FIGURE 2:**
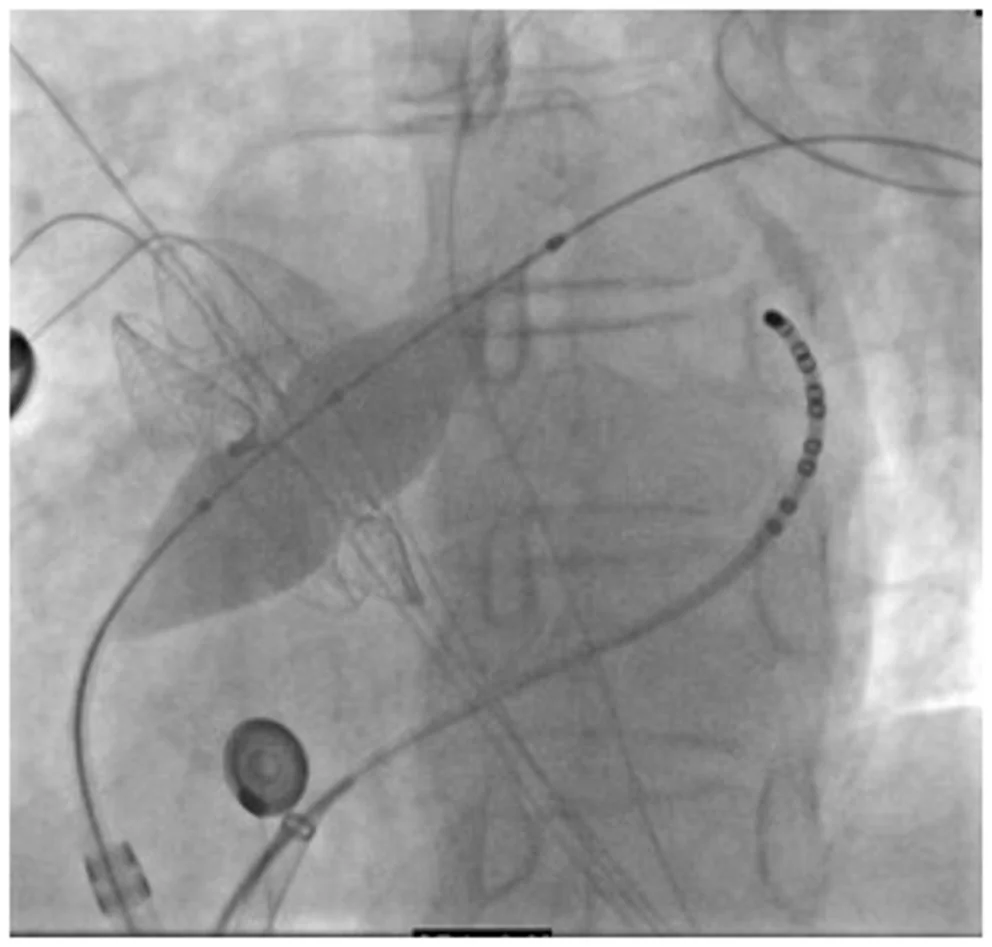
Balloon dilatation of the atrial septal defect (ASD) device using an 18‐mm compliant balloon.

After discussion with the adult congenital heart disease team, balloon dilatation of the device tract was undertaken as a bailout step to overcome device‐mesh recoil and permit passage of the required delivery sheath. An 18‐mm compliant balloon was inflated briefly to nominal pressure for approximately 20 s under fluoroscopic and TOE monitoring. This enabled advancement of the FARADRIVE sheath into the left atrium (Figure 2). Device stability, pericardial effusion, and residual interatrial flow were reassessed immediately after dilatation.

**FIGURE 3:**
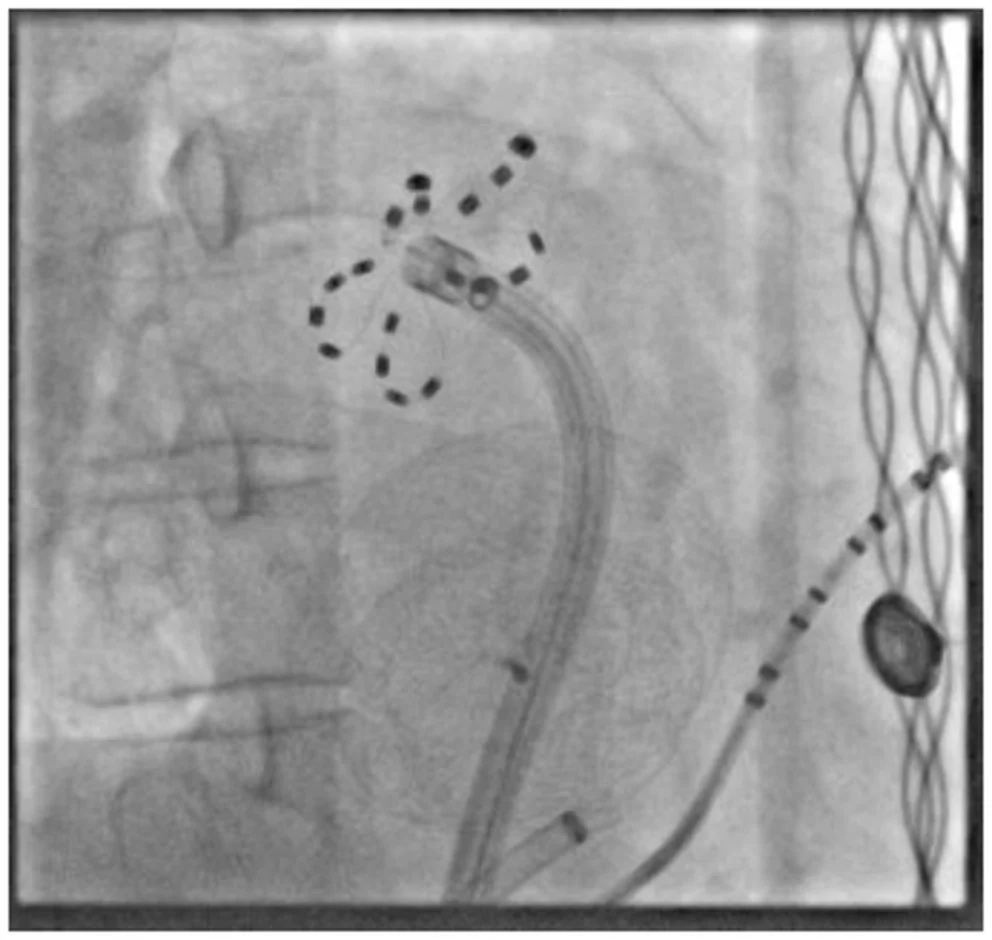
Successful LA access through the ASD closure device, delivering pulsed field ablation (PFA) to the left upper pulmonary vein.

**FIGURE 4:**
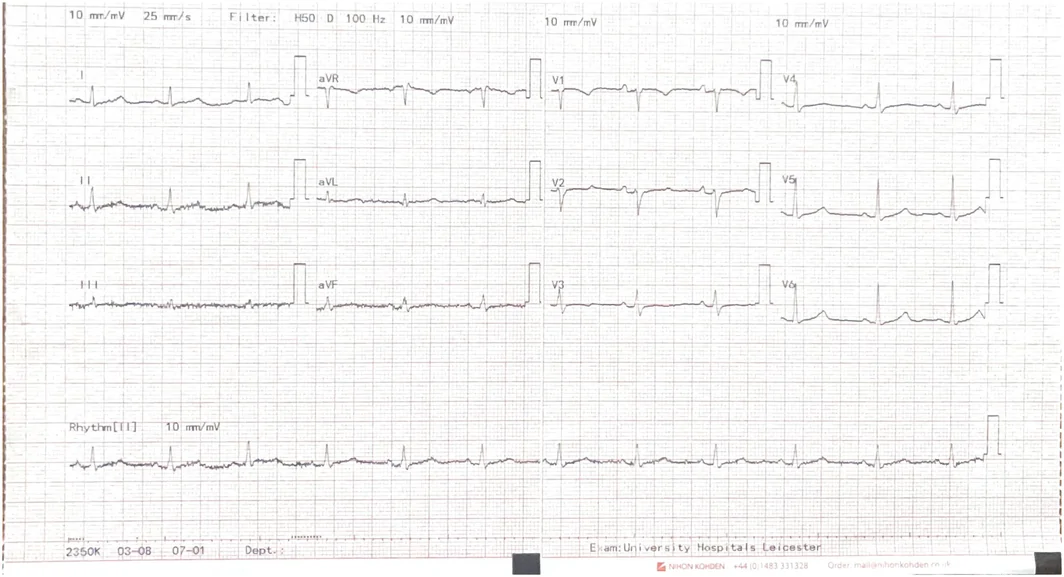
Post‐procedural ECG showing restoration of sinus rhythm.

PFA was then delivered using the FARAWAVE catheter (Figure 3). Energy delivery was guided primarily by fluoroscopy, with TOE available to assess the septal‐device relationship and complications. The catheter was deployed only after confirming an appropriate spline configuration and ensuring it did not contact the ASD device. PFA was performed under fluoroscopic guidance to achieve pulmonary vein isolation (PVI) at the PV ostia and at the left atrium (LA) posterior wall. Six “flower” and four “basket” applications were delivered per superior vein, and four of each per inferior vein. A 200 J DCCV restored sinus rhythm (Figure 4). TOE at the end of the procedure showed no pericardial effusion or device migration but demonstrated residual flow across the septum at the device site. Anticoagulation with Apixaban was maintained throughout the peri‐procedural period. Intravenous unfractionated heparin was administered after transseptal access, with the dose adjusted to maintain an ACT above 300 s, as per current guidelines.

### 2.2 Follow‐Up

At 6 months, the patient reported marked symptomatic improvement, with only mild exertional breathlessness and no recurrence of palpitations or dizziness. Follow‐up TOE at 10 months confirmed sinus rhythm, normal left ventricular (LV) size and systolic function, and a mildly dilated RV with preserved function. A left‐to‐right shunt was noted (Qp:Qs 1.4). This finding prompted multidisciplinary discussion with the adult congenital heart disease (ACHD) team regarding further management and the potential for re‐closure, given the borderline shunt and RV dilatation. It was agreed to proceed with additional shunt assessment in view of the patient's ongoing symptomatic improvement. Cardiac MRI at 13 months showed normal LV size and function, mildly dilated RV with preserved systolic function, and a small residual shunt (Qp:Qs 1.23). Following repeat MDT review, conservative management with clinical follow‐up was advised. At 20‐month follow‐up, the patient remained in sinus rhythm with sustained symptomatic improvement. (Table 1) lists the full case timeline.

**TABLE 1:** Clinical timeline.

Time point	Clinical event
2018	Transcatheter ASD closure using a 33‐mm Figulla Flex II device. Symptoms of dizziness and exertional breathlessness improved after closure.
2 and 10 months after ASD closure	Follow‐up echocardiography showed the device in situ with no residual shunting. The RV remained mildly to moderately dilated with preserved systolic function.
Approximately 2020–2023	Development/progression of symptomatic AF, with fatigue, palpitations, breathlessness, dizziness, and decline in exercise capacity. Two DCCV attempts on amiodarone were unsuccessful.
2023	Referral to arrhythmia clinic for persistent symptomatic AF and consideration of rhythm‐control therapy.
February 2023	Echocardiography confirmed ASD device in situ, preserved biventricular function, and no major interval abnormality.
February 2024	Index procedure: TOE‐guided transseptal access across the ASD device, balloon‐assisted dilatation of the device tract, and PFA pulmonary vein isolation/posterior wall ablation using the FARAWAVE catheter. SR restored with 200 J DCCV.
6 months post‐procedure	Marked symptomatic improvement with no recurrence of palpitations or dizziness.
10 months post‐procedure	TOE confirmed SR and demonstrated left‐to‐right residual shunt, Qp:Qs 1.4, with mildly dilated RV and preserved function.
13 months post‐procedure	CMR showed mildly dilated RV with preserved systolic function and smaller residual shunt, Qp:Qs 1.23. ACHD MDT advised conservative management.
20 months post‐procedure	Patient remained in SR with sustained symptomatic improvement.

In a patient with a large ASD closure device and persistent AF, balloon dilatation of the device tract safely enabled transseptal access for PFA, resulting in successful pulmonary‐vein isolation, restoration, and maintenance of sinus rhythm. Serial imaging demonstrated regression of a borderline residual shunt with preserved right‐sided function, supporting conservative management. This case reinforces that, with imaging guidance and multidisciplinary planning, PFA can be performed across occluder devices and may offer durable clinical benefit in complex congenital anatomy.

## 3 Discussion

ASD is a common congenital heart condition associated with complications such as AF, particularly in adults with repaired or unrepaired defects. AF development in ASD patients results from atrial structural and electrical remodeling secondary to chronic volume overload and atrial stretch. These changes predispose to arrhythmias, even after defect closure, as demonstrated in this case [1].

The European Society of Cardiology (ESC) guidelines emphasize individualized management of AF in patients with congenital heart defects. Rhythm control is recommended for symptomatic patients, especially those with persistent AF, as restoring sinus rhythm can improve quality of life and functional capacity. This is particularly relevant in younger patients or those with significant structural heart disease [3].

Our patient's pre‐closure palpitations and dyspnea suggested long‐standing atrial electrical vulnerability. Her progression to persistent AF post‐device and failure of medical therapy justified the decision for catheter ablation, in line with ESC recommendations [2, 4].

This case highlights the need for careful individualization of transseptal strategy in patients with large septal occluder devices [5, 6]. Published experience supports a stepwise approach: first assessing whether native septal puncture is feasible, and, when direct device puncture is required, using echocardiographic and fluoroscopic guidance with progressive dilatation only when sheath passage fails. In most reports, smaller balloons are used to facilitate tract creation and sheath passage. Therefore, balloon dilatation should not be viewed as routine or risk‐free, particularly when large‐bore sheaths are required. In the present case, dilatation was performed only after failure of standard sheath advancement, with ACHD input and immediate imaging reassessment. This should be interpreted as a bailout maneuver in a highly selected patient, rather than a general recommendation for large‐balloon dilatation across ASD occluder devices [7, 8].

PFA provided a clear procedural advantage in this complex scenario. Its selective electroporation mechanism minimizes collateral injury to adjacent tissue, making it particularly suitable for patients with complex anatomy and prior intracardiac devices [11]. The choice of PFA platform also requires consideration in this setting. The FARAWAVE/FARADRIVE system was selected because it was the locally available and operator‐familiar PFA platform at the time of the procedure, with an established clinical evidence base and a workflow suitable for PVI. However, this advantage must be balanced against the need for a relatively large delivery sheath. Newer or smaller‐profile PFA platforms may reduce the access challenge in future cases and should be considered where available. Similarly, RF ablation remains a reasonable alternative because it can be performed through smaller steerable sheaths, while cryoballoon ablation may offer a single‐shot approach but still requires large‐bore transseptal access and may be less adaptable in complex septal anatomy. The optimal strategy should therefore be determined by device anatomy, residual septal tissue, available technology, operator experience, and ACHD/EP multidisciplinary planning [9].

In our case, TOE guidance was used to delineate the relationship of the closure device to the interatrial septum. Alternative strategies, such as intracardiac echocardiography (ICE) to assist with puncture site selection or the use of specialized steerable and needle‐based puncture sheaths, were considered before proceeding to balloon dilatation. However, given the limited residual septal tissue and repeated difficulty advancing large‐bore sheaths, these options were deemed unlikely to succeed. Balloon dilatation was therefore undertaken as a stepwise escalation, consistent with previously reported techniques, to enable safe passage of the delivery sheath.

A theoretical concern when using PFA in the presence of intracardiac prosthetic material is mechanical interaction between the catheter and the device, and potential electrical interaction if energy is delivered in proximity to metallic structures [9, 11]. In this case, PFA applications were delivered at the pulmonary vein ostia and posterior wall, not adjacent to the ASD occluder. Catheter deployment and spline configuration were assessed before applications, and energy was not delivered if there was concern regarding device contact or constrained deployment. No arcing, catheter instability, device movement, thrombus, or acute haemodynamic compromise was observed. Nevertheless, this remains an important procedural consideration and supports the use of careful imaging, avoidance of energy delivery close to occluder material, and a predefined contingency strategy.

Residual interatrial shunting is an anticipated concern after deliberate dilatation of an ASD occluder tract. In this case, TOE at 10 months demonstrated a left‐to‐right shunt with Qp:Qs 1.4, prompting ACHD MDT discussion regarding re‐closure versus surveillance. The subsequent CMR at 13 months showed a smaller residual shunt, Qp:Qs 1.23, with preserved right‐sided systolic function and sustained symptomatic improvement. In the absence of a hemodynamically significant shunt, progressive RV dilatation, right‐sided dysfunction, cyanosis, paradoxical embolic events, or worsening symptoms, conservative management was considered appropriate. The observed reduction in shunt magnitude may reflect recoil of the nitinol mesh, local tissue apposition, fibrin/thrombus sealing, and progressive endothelialization or fibrotic healing of the device tract. Continued clinical and imaging follow‐up remains important [10, 12].

## Author Contributions

**Ahmed Abdelrazik:** conceptualization, data curation, funding acquisition, methodology, resources, writing – original draft. **Ibrahim Antoun:** formal analysis, project administration, visualization, writing – original draft, writing – review and editing. **Ivelin Koev:** conceptualization, data curation, methodology, writing – review and editing. **Mahmoud Eldesouky:** data curation, resources, writing – review and editing. **Marinos Kantzis:** data curation, supervision, validation, writing – review and editing. **G. Andre Ng:** conceptualization, methodology, project administration, supervision, validation, writing – review and editing.

## Funding

This research was supported by the National Institute for Health Research (NIHR) Leicester Biomedical Research Centre (BRC). NIHR203327.

## Ethics Statement

Formal institutional review board approval was not required for this single‐patient case report in accordance with local legislation and institutional policy. The case was conducted in accordance with the principles of the Declaration of Helsinki.

## Consent

Written informed consent was obtained from the patient for the procedure and for publication of this case report, including the use of anonymized clinical data and images.

## Conflicts of Interest

The authors declare no conflicts of interest.

## Data Availability

The de‐identified clinical data supporting this case report are available from the corresponding author upon reasonable request and may be made available to the journal editors and reviewers for the purposes of editorial or peer‐review assessment, subject to patient confidentiality, consent, and institutional information governance requirements.
